# Early clinical experience with ‘splenic switch-off' in adenosine stress CMR

**DOI:** 10.1186/1532-429X-17-S1-P91

**Published:** 2015-02-03

**Authors:** Jennifer Bryant, Ausami Abbas, Stephen Harden, Steve George, James Shambrook, Charles Peebles

**Affiliations:** University Hospital Southampton NHS Foundation Trust, Southampton, UK

## Background

MRI Adenosine stress perfusion is a well-established method of evaluating myocardial ischaemia but we know from a number of studies that false negatives are a potential weakness of this modality. Assessment of splenic perfusion at stress and rest (splenic switch-off) has recently been suggested as a means of identifying true pharmacological stress response to adenosine[1]. This is a promising technique but can only be assessed *after* the stress procedure. The aim of this study was to compare symptomatic and haemodynamic response with visual assessment of splenic perfusion during stress and rest perfusion imaging to see if we could accurately predict those who would have absent splenic switch-off.

## Methods

We retrospectively reviewed all patients who completed a standard adenosine perfusion CMR over 2 months at a single centre. Adenosine was administered at a standard dose of 140 μg/kg/min for at least 3 minutes prior to perfusion imaging. Adequate haemodynamic response was considered as a heartrate increase ≥ 10bpm and/or SBP decrease ≥ 10 mmHg. Appropriate symptomatic response included facial flushing, breathlessness and chest tightness. According to current clinical practice concurrent haemodynamic and symptomatic response was taken to indicate adequate physiological stress. Splenic switch-off was assessed by visual comparison between stress and rest images.

## Results

145 patients attended for stress. All patients exhibited a response to adenosine. The spleen was not visible on the images of 4 patients (2.8%). Patients were divided into two categories; those who had a single response - haemodynamic ***or*** symptomatic; and those who had a dual response - ***both*** haemodynamic and symptomatic (Table 1).Splenic switch-off was visualized in one of 3 (33%) who had a haemodynamic response only and in 16 of 26 (62%) who had a symptomatic response only. Patients experiencing both a haemodynamic and symptomatic response were more likely to reflect adequate stress with splenic switch-off when compared with those experiencing a single response (p<0.001).

**Table 1:** Splenic switch-off related to adenosine response

			Response to adenosine		
			Single	Dual	Total
Splenic switch-off	NO	Number (%)	12 (41.4%)	7 (6.3%)	19 (13.5%)
	YES	Number (%)	17 (58.6%)	105 (93.8%)	122 (86.5%)
Total	Number (% of total)		29 (20.6%)	112 (79.4%)	141 (100%)

## Conclusions

Using splenic switch-off as the gold standard assessment for adequate pharmacological response there was a good correlation with those who experienced both a haemodynamic and symptomatic response. We should consider increasing adenosine dose when patients show only a single haemodynamic or symptomatic response.

## Funding

N/A.
